# Children's Afterschool Culinary Education Improves Eating Behaviors

**DOI:** 10.3389/fpubh.2022.719015

**Published:** 2022-04-27

**Authors:** Susanne Schmidt, Martin W. Goros, Jonathan A. L. Gelfond, Katherine Bowen, Connie Guttersen, Anne Messbarger-Eguia, Suzanne Mead Feldmann, Amelie G. Ramirez

**Affiliations:** ^1^Department of Population Health Sciences, UT Health San Antonio, San Antonio, TX, United States; ^2^Culinary Health Education for Families (CHEF), San Antonio, TX, United States; ^3^Institute for Health Promotion Research, UT Health San Antonio, San Antonio, TX, United States

**Keywords:** afterschool culinary education, children, effect sizes, evaluation, Latinos, nutrition

## Abstract

**Objective(s):**

Culinary education may be one way to improve children's eating behaviors. We formatively evaluated the effect of a hands-on afterschool 12-module, registered dietitian-led culinary education program on healthy eating behaviors in a predominately Hispanic/Latino, low-socioeconomic community.

**Methods:**

Of 234 children participating in the program, 77% completed both pre- and post-assessment surveys (*n* = 180; mean age 9.8 years; 63.3% female; 74.3% Hispanic/Latino, 88.4% receiving free/reduced lunch). In addition to program satisfaction, we assessed changes in children's self-reported fruit, vegetable, and whole-grain consumption, knowledge, and culinary skills using binary and continuous mixed effects models. We report false discovery rate adjusted *p*-values and effect sizes.

**Results:**

95.5% of participants reported liking the program. Improved whole grain consumption had a medium effect size, while effect sizes for whole grain servings and vegetable consumption were small, but significant (all *p* < 0.05). Culinary skills increased between 15.1 to 43.4 percent points (all *p* < 0.01), with medium to large effect sizes.

**Conclusion(s):**

The program was well-received by participants. Participants reported improved eating behaviors and culinary skills after program completion. Therefore, this hands-on afterschool culinary education program can help improve healthy eating in a predominantly Hispanic/Latino, low-socioeconomic community.

## Introduction

The US has seen an emerging public health challenge with the rise of childhood obesity (1). Nationally, 18.5% of 2 to 19 year olds were obese based on data from the 2015–2016 National Health and Nutrition Examination Survey, with Hispanic youth having significantly higher rates of obesity compared to non-Hispanic White youth (24.8% vs. 14.1%) (2). In Texas, 15.5% of 10–17 year olds were children with obesity based on recent data from the 2017–18 National Survey of Children's Health (3). Hispanic and non-Hispanic youth were not significantly different in Texas [16.7% (Confidence Interval CI: 10.8–24.8) vs. 9.8% (CI: 6.4–14.9)] (4). However, one study examining trends and disparities in childhood obesity in South Texas from 2009 to 2015 found levels of obesity exceeding national averages as well as significant racial/ethnic disparities (5). As obesity is linked to an increased risk of a number of obesity related comorbid conditions (6), prevention of obesity in children is paramount.

One first step in curbing childhood obesity and reducing its negative health effects, is to address childhood nutrition and culinary education (7, 8). Several programs for children have been developed and tested in different settings (school, community, afterschool) for different age groups and target populations. Some programs incorporate exercise interventions (9, 10), others focus on bringing trained chefs into schools to improve attitudes toward fruits and vegetables and increase healthy eating self-efficacy (11–13) or using gardening experiences jointly to improve preferences and willingness to try healthy foods (14). Evaluation of these studies have demonstrated promising results. For instance, a randomized controlled assessment of a 4-h cooking with kids program among fourth graders found that significantly improved fourth-grade students' vegetable preferences, attitudes and self-efficacy among participants compared to the control group (11). However, participants were predominately non-Hispanic white and the study did not assess changes in dietary intake. A randomized controlled trial in a largely Hispanic community combined gardening, nutrition, and cooking education in a 12-week program to successfully improve knowledge and self-efficacy among 3rd through 5th graders (14). Another study among third to eighth graders using a one-group pre-test/post-test evaluation design demonstrated significantly improved nutrition knowledge, cooking self-efficacy, vegetable consumption, and communication about healthy eating 6 months after the end of the 10-week course (13). The study focused on socioeconomically disadvantaged students and had a high participation rate of Hispanic and African American students. Another culinary and nutrition program in an afterschool setting had promising results for reducing BMI and increasing children's desire to cook at home (15, 16). While implemented with predominately minority children, the overall sample sizes were small and the studies reported few significant effects highlighting the need for further investigation.

The CHEF Bites Curriculum is a registered dietitian led hands-on 12-module culinary education program that incorporates nutrition education, culinary skills demonstration and practices, and guided food tasting. This program is novel as it was designed specifically to (1) include nutrition programming that is basic, friendly, unintimidating and relevant to children and their caregivers; (2) integrate culinary skills training into nutrition lessons for younger and older children; (3) incorporate ingredients and modified recipes that are culturally relevant to South Texas; (4) build on a curriculum that allows children to be physically engaged in the nutrition and culinary lesson; (5) engage children based on priorities important to them (i.e., how they will feel, their energy, their ability to be alert, do well in school rather than emphasizing words like “healthy”); and (6) include a bilingual curriculum and bilingual recipes. Our aim was to formatively evaluate the effect of this culturally sensitive, hands-on culinary education program delivered in English at a Boys and Girls Club on eating behaviors, knowledge, and culinary skills of children from predominately Hispanic/Latino and low-socioeconomic communities and to estimate effect sizes for future planning.

## Materials and Methods

### Intervention and Implementation

A team of registered dietitians, culinary professionals, and curricula specialists developed and refined the CHEF Bites Curriculum. Social Cognitive Theory (17) served as the theoretical framework to guide the design of the class structure, and lesson content. Lessons required the instructor to model culinary skills and healthy eating to not only create the initiation of a new behavior, but also to promote maintenance of healthy habits including increased fruit, vegetable, and whole grain consumption, and cooking more at home. The lesson objectives aimed at fostering healthy eating behaviors among participants. Additionally, the team incorporated aspects of the Health Belief Model (18) to break down the perceived barriers to healthy eating and cooking. Lesson materials promoted healthy eating as a way to prevent disease in order to encourage behavior change. CHEF Bites has 12 modules of nutrition and cooking skill education led by a registered dietitian. Each module consists of education on a culinary/nutrition topic, demonstration of skills and a recipe, hands-on cooking by participating children, as well as family-style tasting of the recipes. The modules taught include “Cook Well, Eat Well, Live Well”; “Go With Nature”; “Building Flavor with Herbs and Spices,” “Taste and See a Rainbow of Fruits and Vegetables”; “Power Up with Breakfast”; “Rethink Your Drink”; “Smart Snacks”; “Discovering Foods of the World”; “Dulce De Health”; “The Great Garden Experience-Enjoying Seasonal Foods”; “Sports Nutrition”; and “The Good, the Bad, and the Ugly – Cooking with the Healthiest Dietary Fats.” These modules addressed the basics of healthy eating and cooking; the importance of a healthy breakfast; how to use herbs and spices; choosing healthy drinks and creating infused water; reading nutrition labels and identifying and making healthier snacks and treats; basics of sport nutrition and healthy fats. A registered dietitian and culinary professional guided the development of the curriculum and recipes. The program is culturally sensitive and includes flavors of the local cuisine. The curriculum focuses on five tenets for healthy eating and living: (1) Eat a healthy breakfast; (2) Drink plenty of water; (3) Fill half their plate with fruits and vegetables; (4) Eat mostly plant-based, less processed foods; and (5) Cook and eat at home. The curriculum was piloted at the Boys and Girls Club prior to the formative evaluation. Classes included nutrition education, culinary skill demonstration, hands-on skill practice by children, and dietitian-guided, family-style sit down tasting.

For the evaluation of the program at the Boys and Girls Club, three implementations took place as afterschool programs with once-a-week classes over a 3-month period and one implementation took place over the summer with twice-a-week classes over an 8-week period for a total of 12 modules at the same teaching kitchen. Each module was ~60 min but varied based on module content and recipe used. Programming was similar across implementation cycles with changes in the order of class modules. Class instructors reported high fidelity with 92% of modules taught as outlined in the curriculum. If classes had to be canceled due to illness, holidays, or other reasons, the instructor offered make-up classes. There was no additional cost for children to participate in the culinary education program besides the regular annual or summer membership fee.

### Design of Survey Instruments

For the purpose of evaluation, we developed pre- and post-assessment surveys for children participating in the program to complete before and after receiving programming in a pre-test/post-test within subjects design. Surveys assessed eating behaviors, culinary skills, and nutrition knowledge as well as program satisfaction (see Supplementary Materials). Further, children completed a short after-class survey after each module to assess attendance as well as satisfaction with the module and the recipe. Instrument development was guided by a review of existing tools (19, 20) and tailored specifically to the evaluation needs of the program and the target population. The instruments were evaluated for readability and were tested with four children from the Boys and Girls Club of the target age group. During the first round of implementation, we observed pre-assessment survey administration identifying no major difficulties with children completing the surveys. Surveys were collected during four cycles of implementation in 2018 and 2019.

### Participants

Inclusion criteria for the evaluation study were that children had to be able to speak English and read and write in English. The recommended ages for participation were 9 to 13 years old to ensure children would be able to complete the assessments and to maximize retention as older children had more flexibility in selecting and attending afterschool activities. As these ages were recommendations only, some younger and older children also participated. Participants were selected by the Boys and Girls Club based on these recommended inclusion criteria. The Boys and Girls Club provided us with children's sociodemographic information from their annual registration forms. The Boys and Girls Club has multiple branches across the city. One branch has a fully equipped teaching kitchen. Children from the branch with the teaching kitchen as well as children from other branches were able to participate in the program through transportation provided by the Boys and Girls Club. A total of 234 school-aged children completed the pre-assessment survey before participating in the program. At the end of the program, 180 children had completed both the pre- and post-assessment surveys.

### Data Collection

All evaluation data were collected and managed using REDCap electronic data capture tools hosted at UT Health San Antonio (21, 22). Evaluation staff trained local staff and instructors on how to administer the survey to children using tablets. To help participants estimate serving sizes of fruits, vegetables and whole grains, children had access to serving size food replicas that they could pick up while taking the pre- and post-assessment survey. Our evaluation project was reviewed by the UT Health San Antonio Institutional Review Board and deemed to be a non-research activity. Parents/caregivers of participating children agreed when enrolling children at the Boys and Girls Club that children were allowed to be observed during program implementation and that children were allowed to complete surveys for the purpose of program evaluation.

### Outcome Measures

We assessed behaviors at baseline and after completion of the program using a variety of measures. First, children were asked whether they consumed fruits, vegetables, whole grains, and sugar-sweetened beverages on the day prior to the survey If they indicated eating or drinking these food types/beverages, they were asked how many servings they had consumed using food replicas as aids for estimation. Children were further asked how often children participated in buying groceries, making food choices for the family, and speaking with their families about healthy food. We also assessed nutrition and culinary knowledge and skills through items that asked whether children had ever heard of MyPlate, whether children were able to identify all five tenets of the program correctly, and through several self-reported cooking skills.

### Data Analysis

To show the sociodemographic composition of our participants, we calculated descriptive univariate statistics for (1) all children who completed the pre-assessment (*n* = 234), (2) those who only had pre-assessment surveys (*n* = 54), and (3) those who completed both pre-and post-assessment surveys (*n* = 180). We also report *p*-values based on Chi-Squared tests comparing those who only completed the pre-assessment and those who completed both pre- and post-assessments.

Estimates and *p*-values for behaviors, knowledge, and skills are based on binary or continuous mixed effects models depending on the outcome. These models accounted for within subject correlations (pre/post) and the implementation cycle in which children were enrolled. Additionally, we calculated false discovery rate (FDR) adjusted *p*-values accounting for testing of the same dataset for multiple outcomes (23). We estimated effect sizes using Cohen's *d* (continuous outcome) and *g* (binary outcome) as a supplement to our significance tests to provide measures of effect magnitude for future study planning (24, 25). Effect sizes greater or equal to 0.2 and 0.05 are considered non-trivial using Cohen's *d* (24) and Cohen's *g* (25), respectively. All analyses were conducted using R (R Foundation for Statistical Computing, Vienna, Austria).

## Results

### Sociodemographic Characteristics and Program Satisfaction

At total of 234 children participated in the culinary program in four rounds of implementations in 2018 and 2019. Approximately 77% of children (*n* = 180) completed both pre- and post-assessment surveys. Participating children were predominately female, Hispanic/Latino, lived in single parent households, and received free or reduced lunch at school (Table 1). Children who only took the pre-assessment survey were more likely to be older, males, Hispanic/Latino, and had free/reduced lunch compared to those who completed both surveys (*p* < 0.05 for all). Hispanic/Latino children were 3 times more likely to only complete a pre-assessment survey compared to non-Hispanic/non-Latino children (*p* = 0.029). All children (*n* = 234) enrolled in the program attended on average 8.8 of the 12 classes, with 74.4% of children attending 8 or more classes. On average, children who completed the post-assessment survey (*n* = 180) attended 9.7 classes, with 87.8% of children attending at least 8 classes.

**Table 1:** Children's sociodemographic characteristics, program participation, and program satisfaction of children who completed the pre-assessment survey, those who completed both the pre- and post-assessment surveys and those who only completed the pre-assessment survey.

	**Completed pre-assessment** ***N* (%)**	**Completed pre-assessment only** ***N* (%)**	**Completed pre- and post-assessment** ***N* (%)**	***p*-value**
Number of children	234	54	180	
Implementation cohort				0.300
Spring 2018	54 (23.1)	14 (25.9)	40 (22.2)	
Summer 2018	62 (26.5)	9 (16.7)	53 (29.4)	
Fall 2018	57 (24.4)	14 (25.9)	43 (23.9)	
Spring 2019	61 (26.1)	17 (31.5)	44 (24.4)	
Age				0.008
Mean (sd)	9.91 (1.32)	10.33 (1.50)	9.79 (1.23)	
Range	7–16	8–16	7–15	
Gender				0.046
Female	140 (59.8)	26 (48.1)	114 (63.3)	
Male	94 (40.2)	28 (51.9)	66 (36.7)	
Ethnicity				0.024
Hispanic/Latino	187 (79.9)	49 (90.7)	138 (76.7)	
Non-Hispanic/Latino	47 (20.1)	5 (9.3)	42 (23.3)	
School meal status				0.042
Receives free/reduced lunch	202 (89.8)	49 (98.0)	153 (88.4)	
Does not receive free/reduced lunch	21 (9.3)	1 (2.0)	20 (11.6)	
Missing	9	2	7	
Lives in single parent household				0.144
Yes	141 (64.7)	36 (73.5)	105 (62.1)	
No	77 (35.3)	13 (26.5)	64 (37.9)	
Missing	16	5	11	
Number of classes attended				<0.001
Mean (sd)	8.76 (2.71)	5.65 (2.62)	9.70 (1.93)	
Range	1–12	1–11	2–12	
Children attending 8 or more classes				<0.001
Yes	174 (74.4)	16 (29.6)	158 (87.8)	
No	60 (25.6)	38 (70.4)	22 (12.2)	
Overall program satisfaction				
Liked program a lot	–	–	148 (82.2)	
Liked program	–	–	24 (13.3)	
Unsure	–	–	6 (3.3)	
Did not like program	–	–	0	
Did not like program at all	–	–	2 (1.1)	
Would recommend CHEF program to friends				
Yes	–	–	139 (77.2)	
Maybe	–	–	37 (20.6)	
No	–	–	4 (2.2)	

*sd, Standard deviation; P-value comparing children who completed pre-assessment only and children who completed pre- and post-assessments*.

During the post-assessment survey, we asked children how well they liked the classes overall and whether they would recommend the program to their friends. The program was very well-received. As such, 82.2% of all children who completed the survey stated that they liked the classes a lot, with an additional 13.3% of children stating that they liked the classes overall. In addition to high overall satisfaction, 77.2% of all participants stated that they would recommend the culinary program to their friends. Program satisfaction was similar across all implementation cycles.

### Changes in Behavior

We saw significant increases in the percent of children stating that they ate vegetables and whole grains (Table 2). At baseline, 55.6% of children stated that they had eaten vegetables the day before the survey compared to 68.3% of children during post-assessment (FDR adjusted *p* = 0.02). Further, 49.4% of children indicated eating whole grains the day before the baseline survey compared to 68.3% of children during post-assessment (FDR adjusted *p* < 0.001). Participants also significantly increased the number of servings of vegetables (FDR adjusted *p* = 0.028) and whole grains (FDR adjusted *p* < 0.001) that they consumed. The change in servings of fruit was significant using traditional *p*-values but not significant using FDR adjusted *p*-values. There was a small improvement toward fewer sugary beverages consumed, but this trend was *not* significant. We saw no changes in eating breakfast or preparing and eating dinner at home. During post-assessment, 76.7% of children stated that they felt the program had changed the way they ate at home and 81.7% stated that they now liked new foods because of participating in the program. We further assessed how often children participated in buying groceries, making food choices for the family, and speaking with their families about healthy food. Children stated being more involved in picking out foods eaten at home in the post-assessment survey compared to the pre-assessment survey (FDR adjusted *p* = 0.01). Participants also reported talking more frequently with their family about eating and cooking healthy food (FDR adjusted *p* = 0.005). While we saw a positive trends toward more children indicating going grocery shopping with their family, this trend was not significant in our cohort of children participating in the culinary program at the Boys and Girls Club in 2018–2019. Effect sizes for eating whole grains were in the medium range, while effect sizes for servings of whole grains, vegetable consumption, making food choices for the family and speaking with their family about healthy were small, but significant. The effect size for vegetable servings was too small and is considered to be trivial.

**Table 2:** Changes in eating behaviors in children who completed both pre- and post-assessment surveys (*n* = 180).

	**Pre-assessment** ***N* (%)**	**Post-assessment** ***N* (%)**	***p*-value**	**FDR adjusted** ***p*-value**	**Effect size**
Ate vegetables yesterday (a)			0.01	0.02	0.14
Yes	100 (55.6)	123 (68.3)			
No	80 (44.4)	57 (31.7)			
Ate fruit yesterday (a)			0.11	0.149	0.11
Yes	136 (75.6)	148 (82.2)			
No	44 (24.4)	32 (17.8)			
Ate whole grains yesterday (a)			<0.001	<0.001	0.22
Yes	89 (49.4)	123 (68.3)			
No	91 (50.6)	57 (31.7)			
Drank sugary drinks yesterday (a)			0.32	0.358	−0.07
Yes	121 (67.2)	113 (62.8)			
No	59 (32.8)	67 (37.2)			
Servings of vegetables (b)			0.02	0.028	0.18
0 servings	80 (44.4)	57 (31.7)			
1 serving	40 (22.2)	51 (28.3)			
2 servings	44 (24.4)	49 (27.2)			
3 or more servings	16 (8.9)	23 (12.8)			
Servings of fruit (b)			0.04	0.051	0.16
0 servings	44 (24.4)	32 (17.8)			
1 serving	60 (33.3)	55 (30.6)			
2 servings	46 (25.6)	51 (28.3)			
3 servings	13 (7.2)	25 (13.9)			
4 servings	10 (5.6)	5 (2.8)			
5 or more servings	7 (3.9)	12 (6.7)			
Servings of whole grains (b)			<0.001	<0.001	0.33
0 servings	91 (50.6)	57 (31.7)			
1 serving	41 (22.8)	51 (28.3)			
2 servings	34 (18.9)	36 (20.0)			
3 or more servings	14 (7.8)	36 (20.0)			
Servings of sugary drinks (b)			0.08	0.117	−0.13
0 servings	59 (32.8)	67 (37.2)			
1 drink	66 (36.7)	73 (40.6)			
2 drinks	43 (23.9)	29 (16.1)			
3 or more drinks	12 (6.7)	11 (6.1)			
Had breakfast yesterday (a)			0.41	0.441	−0.07
Yes	154 (85.6)	149 (82.8)			
No	26 (14.4)	31 (17.2)			
Last week, cooked and ate dinner at home at least 5 times (a)			0.66	0.66	0.02
Yes	77 (42.8)	81 (45.0)			
No	103 (57.2)	99 (55.0)			
Program changed the eating habits at home			–	–	–
Yes	–	138 (76.7)			
No	–	42 (23.3)			
Like new foods because of the program			–	–	–
Yes	–	147 (81.7)			
No	–	33 (18.3)			
Go to store to buy food with family (b)			0.12	0.155	0.11
Yes—often	84 (46.7)	96 (53.3)			
Yes—sometimes	82 (45.6)	72 (40.0)			
Almost never	8 (4.4)	6 (3.3)			
No	6 (3.3)	6 (3.3)			
Help pick out foods eaten at home with family (b)			0.01	0.01	0.21
Yes—often	55 (30.6)	73 (40.6)			
Yes—sometimes	86 (47.8)	82 (45.6)			
Almost never	16 (8.9)	10 (5.6)			
No	23 (12.8)	15 (8.3)			
Talk with family about eating and cooking healthy food (b)			0.003	0.005	0.22
Yes—often	36 (20.0)	52 (28.9)			
Yes—sometimes	82 (45.6)	81 (45.0)			
Almost never	24 (13.3)	21 (11.7)			
No	38 (21.1)	26 (14.4)			

*(a): Mixed effects model (binary), effect size using Cohen's g.*

*(b): Mixed effects model (continuous), effect size using Cohen's d.*

*FDR, False discovery rate*.

### Changes in Knowledge and Skills

We saw significant improvements in children's knowledge and self-reported culinary skills (Table 3). All culinary skills assessed in the pre- and post-surveys showed significant (FDR adjusted *p* < 0.05) improvements ranging from 15.1 to 43.4 percent point increases in self-reported skills. Further, during the post-assessment, almost all children (92.2%, 166 out of 180) stated that they had heard of MyPlate compared to 53.9% (97 out of 180) during pre-assessment, a statistically significant improvement (FDR adjusted *p* < 0.001). During pre-assessment, 22.2% of children correctly identified all the program's tenets, the five activities they should do to grow healthy and strong (eat a healthy breakfast; drink plenty of water; fill half their plate with fruits and vegetables; eat mostly plant-based foods; and cook and eat at home), compared to 53.3% of children during post-assessment (FDR adjusted *p* < 0.001). Effect sizes for all knowledge and skill measures ranged from |0.25| to |0.49|, indicating medium to large effect sizes using Cohen's *g*.

**Table 3:** Changes in knowledge and skills among children who completed both pre- and post-assessment surveys (*n* = 180).

	**Pre-assessment** ***N* (%)**	**Post-assessment** ***N* (%)**	***p*-value**	**FDR adjusted *p*-value**	**Effect size**
Ever heard of MyPlate (a)			<0.001	<0.001	0.49
Yes	97 (53.9)	166 (92.2)			
No	83 (46.1)	14 (7.8)			
Correct program tenets (a)			<0.001	<0.001	0.36
Yes	40 (22.2)	96 (53.3)			
No	140 (77.8)	84 (46.7)			
**Cooking skills**
Measure ingredients (a)			<0.001	<0.001	0.38
Yes	91 (50.6)	134 (74.4)			
No	89 (49.4)	46 (25.6)			
Chop an onion or other food (a)			<0.001	<0.001	0.37
Yes	101 (56.1)	147 (81.7)			
No	79 (43.9)	33 (18.3)			
Make a salad dressing (a)			<0.001	<0.001	0.38
Yes	38 (21.1)	104 (57.8)			
No	142 (78.9)	76 (42.2)			
Cook rice (a)			<0.001	<0.001	0.44
Yes	41 (22.8)	110 (61.1)			
No	139 (77.2)	70 (38.9)			
Slice a cucumber or other food (a)			<0.001	<0.001	0.42
Yes	100 (55.6)	151 (83.9)			
No	80 (44.4)	29 (16.1)			
Follow a recipe (a)			<0.001	<0.001	0.34
Yes	117 (65.0)	154 (85.6)			
No	63 (35.0)	26 (14.4)			
Use a knife safely (a)			<0.001	<0.001	0.30
Yes	127 (70.6)	158 (87.8)			
No	53 (29.4)	22 (12.2)			
Boil pasta (a)			<0.001	0.001	0.26
Yes	47 (26.1)	74 (41.1)			
No	133 (73.9)	106 (58.9)			
Stir-fry vegetables (a)			<0.001	<0.001	0.43
Yes	31 (17.2)	109 (60.6)			
No	149 (82.8)	71 (39.4)			
None of these (a)			0.002	0.004	−0.25
Yes	20 (11.1)	8 (4.4)			
No	160 (88.9)	172 (95.6)			

*(a): Mixed effects model (binary), effect size using Cohen's g.*

*FDR, False discovery rate*.

## Discussion

Overall, the 2018–2019 hands-on culinary program at the Boys and Girls Club was well-received by participating children. The curriculum, which was designed using social cognitive theory and the health beliefs model, predominately served Latino/Hispanic children and children who received free or reduced lunch at school. For the four implementation cycles, we saw statistically significant improvements in whole grains and vegetable consumption, knowledge of MyPlate and the program's five tenets for healthy eating and living, and self-reported culinary skills as well as changes in talking about healthy eating and cooking and helping pick out foods, with effect sizes ranging from small to large. We saw no statistically significant changes in other outcomes such sugary beverage consumption, eating breakfast or preparing and eating dinner at home. However, a vast majority of children stated that they liked the program and that the CHEF Bites Curriculum had changed the way they ate at home, including liking new foods because of their participation in the program.

Our findings in improved consumption of healthy foods and knowledge are supported by other studies. A study in Chicago among socioeconomically disadvantaged and minority students also reported significantly improved scores for nutrition knowledge, vegetable consumption, and communication about healthy eating (13). Similarly, to our evaluation, the Chicago study used a one-group pre-test/post-test evaluation design. However, the study had a 59% completion rate for pre- and post-assessment surveys (13). Our study, while having a smaller sample size, was able to collect pre- and post-assessment from 77% of children who began the program. A study using a randomized control trial in a largely Hispanic community that included gardening and nutrition education, also found significant improvements in nutrition knowledge and other factors influencing dietary behavior (14). Our study adds to these findings as the majority of our program participants were Hispanic.

While we found improvement in some behaviors, we saw no significant changes in consumption of sugary beverages. Wieland et al. reported positive trends in attitudes and motivation for fruit and vegetable consumption, sugary beverage consumption but these were not statistically different from baseline in their afterschool nutrition and exercise intervention fielded at a Boys and Girls Club (10). The authors cite small sample and timing of data collection as potential reasons for lack of statistical significance. The Wieland study collected baseline surveys in October with greater availability of fruits and vegetables and outdoor exercise opportunities while post-assessment took place in April in Minnesota with limited opportunities for exercise (10). Similarly, timing of our data collection may have affected the assessment of sugary beverage consumption, as participants completed post-assessment surveys at the end of December and late Spring where classroom celebrations (e.g., for Winter and Spring holidays) including sugary beverages and more unhealthy foods may be more common. Future studies should consider the effect of timing on outcomes.

Our study was able to show improvements in healthy eating behaviors immediately following the completion of the program; however, our evaluation was not designed to assess sustained changes in eating behaviors and other outcomes for children. Other studies have included longer-term follow-up to assess sustained changes (13, 26). For instance, one study found sustained improvements in healthy eating communication at 6 months after program completion (13). Future evaluation efforts should include longer-term follow-up to assess whether the CHEF Bites Curriculum has a lasting effect on children's eating behaviors.

This evaluation was based on children's self-reported outcomes only, while other studies have assessed parent-reported changes in child and family outcomes (13), as children live in complex environment and their eating behaviors are influenced by their home environment both through accessibility of healthy foods and lived experiences (27). An evaluation including both children's and parents' perspectives on changes in healthy eating behaviors can shed light on these complex environments and may aid in the development of culinary and nutrition programs aimed at children and their families. Future iterations of the program could also assess changes in cooking skills by observation instead of self-report.

Based on the promising findings presented here, future studies should examine how children perceive the CHEF Bites Curriculum in other settings with or without the hands-on component, for instance in schools without teaching kitchens, or other modes of delivery, for instance registered dietitian vs. community health worker or promotores as instructors. Additionally, future studies with a larger sample of children could examine whether the program works equally well for different age groups and how many classes are needed to achieve meaningful change in behaviors. The calculated effect sizes that we report for all outcomes including eating behaviors, culinary skills, and involvement in making food choices at home may be used for future planning purposes.

### Limitations

We acknowledge the following limitations to this evaluation. (1) We used a one-group pre-test/post-test evaluation design surveying children before and after participating in the program without administering surveys to a control group. (2) The evaluation was not sufficiently powered for subgroup analyses and the program may have different effects on some children based on their sociodemographic characteristics. (3) We did not adjust for how many modules each child completed in our analysis. However, 87.8% of children who completed both surveys attended at least 8 out of 12 classed, which was needed to be considered a program graduate. (4) Approximately 77% of children completed both pre- and post-assessment surveys and children who were lost to follow up were different in age, sex, ethnicity, and school lunch status. This non-random loss to follow-up also poses a limitation as it is possible that children who did not complete the post-assessment had a different experience with the program, resulting in selection bias and reduced generalizability. (5) Our study did not include a systematic follow-up with children to establish longer-term changes in behavior, knowledge, and skills as the study was designed as a formative evaluation. While these limitations exist, the results from this evaluation are promising that hands-on culinary education programs in afterschool and summer program settings can have a positive effect on children's eating behaviors and cooking skills, which—if sustained—can lead to improved health in children.

## Conclusion

Our formative evaluation of the CHEF Bites curriculum, a hands-on, registered dietitian-led culinary education program, implemented at a Boys and Girls Club as an afterschool and summer program improved healthy eating behaviors, knowledge, and self-reported culinary skills in participating children. The evaluation took place in a predominantly Hispanic/Latino, low-socioeconomic community and incorporated healthy versions of dishes inspired by the local cuisine into the curriculum. Cultural appropriateness may be one factor that needs to be considered in designing and implementing successful culinary education programs for children and their families. Children reported becoming more involved in making decisions regarding the food that the family consumed which may have positive effects on their families as a whole.

## Data Availability Statement

De-identified raw outcomes data supporting the conclusions of this article will be made available upon request.

## Ethics Statement

The UT Health San Antonio Institutional Review Board reviewed our program evaluation and deemed it to be a non-research activity (Protocol number: HSC20170654N). Separately, parents/caregivers of participating children agreed when enrolling children at the Boys and Girls Club that children were allowed to be observed during program implementation and that children were allowed to complete surveys for the purpose of program evaluation.

## Author Contributions

SS has contributed to all parts of the manuscript, including designing the evaluation plan, data collection, analysis planning, data analysis and interpretation, manuscript writing, and revision. MG conducted the statistical analysis, interpreted the results, contributed to writing, and revising the manuscript. JG contributed to design of the evaluation plan, designed the analysis plan, interpreted the results, contributed to writing, and revising the manuscript. SF and AM-E contributed to the development of the evaluation plan, data collection instruments, writing, and revising the manuscript. CG and KB developed and revised the CHEF Bites Curriculum, provided the theoretical framework for the program, contributed to writing, and revising the manuscript. AR supervised all activities and contributed to the evaluation design, interpretation of results, manuscript writing, and revision. All authors agreed to be accountable for the content of the work, contributed to the article, and approved the submitted version.

## Funding

This project was supported by a contract from CHEF (Culinary Health Education for Families) to UT Health San Antonio to conduct the independent evaluation of the CHEF program at the Boys and Girls Club in 2018 and 2019.

## Conflict of Interest

SF is the CEO and AM-E is the Vice President, Strategy and Operations of the CHEF (Culinary Health Education for Families) program, the program that was evaluated in this manuscript, provided feedback for the development of the evaluation plan and data collection instruments but were not involved in the analysis of the data and remained independent of the evaluation. CG and KB developed and revised the CHEF Bites Curriculum. The remaining authors declare that the research was conducted in the absence of any commercial or financial relationships that could be construed as a potential conflict of interest.

## Publisher's Note

All claims expressed in this article are solely those of the authors and do not necessarily represent those of their affiliated organizations, or those of the publisher, the editors and the reviewers. Any product that may be evaluated in this article, or claim that may be made by its manufacturer, is not guaranteed or endorsed by the publisher.
