# Supplementary material for: Children's Afterschool Culinary Education Improves Eating Behaviors
Source: Front Public Health. 2022 Apr 27;10:719015. doi: 10.3389/fpubh.2022.719015 (PMC9091819; doi:10.3389/fpubh.2022.719015)
Supplement: Supplementary file 1 [file Data_Sheet_1.pdf]

Supplementary Material

CHEF Bites Pre- and Post-Assessment Survey

Participants: Children participating in the Mays Boys and Girls Club CHEF Bites Program

Data collection: Electronical data collection through REDCap

[Pre-Assessment Survey Introduction] We want to learn more about what and how you eat. Please answer the questions truthfully and do your best to remember what you had to eat and drink. This is NOT a test, so there are no right or wrong answers. If there is anything you don't know, use your best guess.

[Post Assessment Survey Introduction] Hi there again. We want to learn more about what and how you eat. Please answer the questions truthfully and do your best to remember what you had to eat and drink. This is NOT a test, so there are no right or wrong answers. If there is anything you don’t know, use your best guess.

1. First Name: _______________
2. CHEF ID#: ___________

3. Did you eat any vegetables yesterday? Vegetables are all cooked and uncooked vegetables (like carrots, peas, broccoli); green salads; boiled, baked and mashed potatoes. Do not count French fries or chips.
   - ☐ Yes
   - ☐ No

SKIP LOGIC IF 3=Yes: How many servings of vegetables did you eat yesterday?

A serving is the size of your fist. Use the serving size examples to help you count.

- ☐ 1 serving
- ☐ 2 servings
- ☐ 3 servings
- ☐ 4 servings
- ☐ 5 or more servings

4. Did you eat any fruit yesterday? Fruits are things like apples, bananas, oranges, grapes. Please do not count any fruit juice.
   - ☐ Yes
   - ☐ No

SKIP LOGIC IF 4=Yes: How many servings of fruit did you eat yesterday? A serving is the size of your fist. Use the serving size examples to help you count.

- ☐ 1 serving
- ☐ 2 servings
- ☐ 3 servings
- ☐ 4 servings
- ☐ 5 or more servings

5. Did you eat any foods with whole grains yesterday? Some examples might be whole wheat or whole grain bread, brown rice, whole wheat pasta, whole grain crackers. Don't count white bread, white rice, or white pasta.

- ☐ Yes
- ☐ No

SKIP LOGIC IF 5=Yes: How many servings of whole grains did you eat yesterday? Use the serving size examples to help you count.

- ☐ 1 serving
- ☐ 2 servings
- ☐ 3 servings
- ☐ 4 servings
- ☐ 5 servings
- ☐ 6 servings
- ☐ 7 or more servings

6. Did you drink sugary drinks like fruit punch, sports drinks like Gatorade, fruit-flavored drinks, regular (not diet) sodas, energy drinks, or sweet tea yesterday?

- ☐ Yes
- ☐ No

SKIP LOGIC IF 6=Yes: How many sugary drinks did you drink yesterday?

- ☐ 1 drink
- ☐ 2 drinks
- ☐ 3 or more drinks

7. Did you eat breakfast yesterday?

- ☐ Yes
- ☐ No

8. Please check each cooking skill you know how to do: (Check all that apply)

- ☐ Measure ingredients
- ☐ Chop an onion or other food
- ☐ Make a salad dressing
- ☐ Cook rice
- ☐ Slice a cucumber or other food
- ☐ Follow a recipe
- ☐ Use a knife safely

☐ Boil pasta
☐ Stir-fry vegetables
☐ None of these

9. Have you ever heard of MyPlate?
☐ Yes
☐ No

SKIP LOGIC IF 9=yes: Look at the picture below. Which one is MyPlate?
☐ Plate A *(Correct MyPlate)*
☐ Plate B

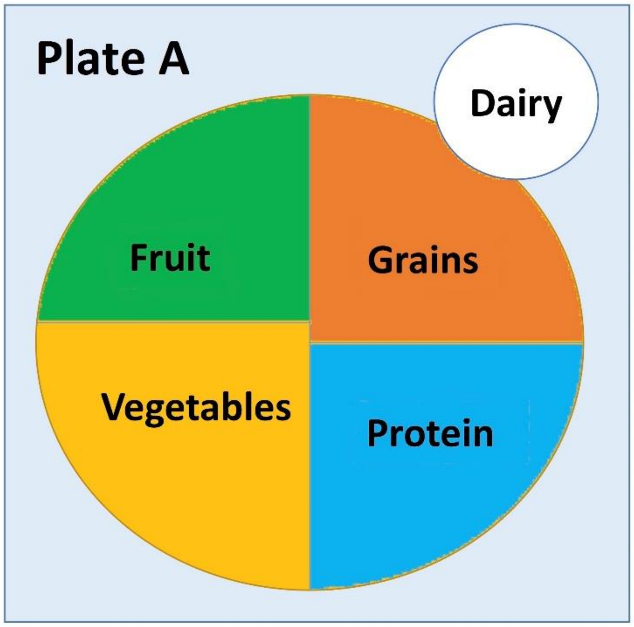

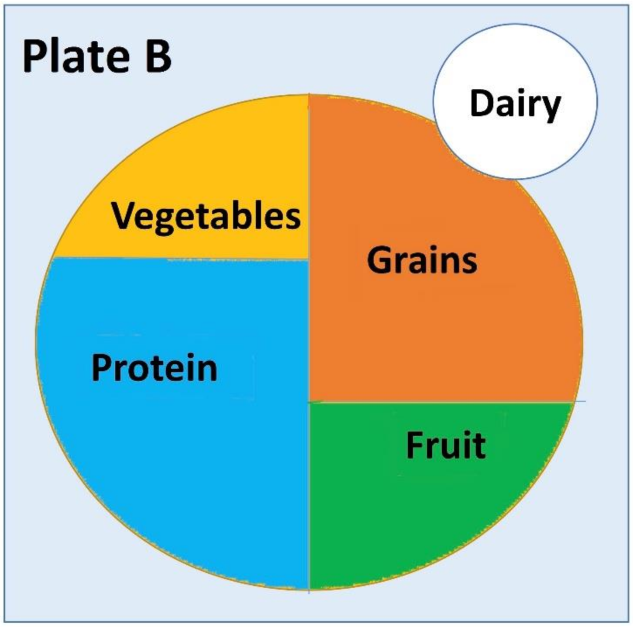

10. How many servings of fruits and vegetables should you be eating every day?
☐ 0 servings
☐ 1 serving
☐ 2 servings
☐ 3 servings
☐ 4 servings
☐ 5 or more servings

11. Which of these drinks is better for you?
☐ Water
☐ Soda/fruit punch

12. Which of these foods is better for you?
☐ White bread
☐ Whole grain or whole wheat bread

13. Which of these foods is better for you?
- ☐ Food fried in fat like French fries or fried chicken
- ☐ Food prepared with less fat like baked potatoes or baked chicken

14. Please check the activities you should do every day to grow up healthy and strong. (Check all that apply)
- ☐ Drink plenty of water
- ☐ Eat dessert
- ☐ Eat a healthy breakfast
- ☐ Drink sports drinks like Gatorade
- ☐ Fill half your plate with fruits and vegetables
- ☐ Eat mostly starchy foods like white rice, white pasta and white bread
- ☐ Eat mostly plant-based foods
- ☐ Drink lots of soda
- ☐ Cook and eat at home
- ☐ Skip breakfast
- ☐ Watch TV while eating your meals

15. When you are at home, who prepares most of the food that you eat?
- ☐ Myself
- ☐ Mom/stepmom
- ☐ Dad/stepdad
- ☐ Grandparent
- ☐ Aunt/uncle
- ☐ Sister/brother
- ☐ Someone else

SKIP LOGIC IF 15=someone else: Please list who prepares most of the food you eat: ______________________

16. Do you go to the store to buy food with your family?
- ☐ Yes – often
- ☐ Yes – sometimes
- ☐ Almost never
- ☐ No

17. Do you help pick out what foods you eat at home with your family?
- ☐ Yes – often
- ☐ Yes – sometimes
- ☐ Almost never
- ☐ No

18. Do you talk with your family about eating and cooking healthy food?
- ☐ Yes – often

- ☐ Yes – sometimes
- ☐ Almost never
- ☐ No

19. Do you feel comfortable talking to your family about healthy food?
- ☐ Yes - I can talk to my family about healthy food and they will listen
- ☐ I can talk to my family about healthy food, but they don't always listen
- ☐ No - my family doesn't listen to me if I talk about healthy food

20. Last week, how often did you or someone else in your family cook food for dinner at home? Do not count food or left-overs from restaurants.
- ☐ 0 (not at all)
- ☐ 1 time
- ☐ 2 times
- ☐ 3 times
- ☐ 4 times
- ☐ 5 times
- ☐ 6 times
- ☐ 7 times (every day)

21. Last week, how often did you eat dinner at home while watching TV or movies, playing video games or playing on a phone or other technology?
- ☐ 0 (not at all)
- ☐ 1 time
- ☐ 2 times
- ☐ 3 times
- ☐ 4 times
- ☐ 5 times
- ☐ 6 times
- ☐ 7 times (every day)

[Questions included in the Post-Assessment Survey ONLY]

22. Has the CHEF class changed how you eat at home?
- ☐ Yes
- ☐ No

SKIP LOGIC IF 22=Yes: What changed about how you eat at home?

____

23. Are there any new foods that you like because of the CHEF classes?
- ☐ Yes
- ☐ No

SKIP LOGIC IF 23=Yes: What new foods do you like now because of CHEF?

____

24. Do you try to eat breakfast every day?
- ☐ Yes, I try and I do most of the time
- ☐ Yes, I try, but it is difficult
- ☐ No, but I will try in the future
- ☐ No, and I don't think I will try

SKIP LOGIC IF 24=Yes, I try and I do most of the time: What do you think helps you be successful at eating breakfast every day? ________________________

SKIP LOGIC IF 24=Yes, but it's difficult: Can you explain what makes it difficult to eat breakfast every day? ____

SKIP LOGIC IF 24=No, but I will try it in the future: What do you think would make it easier for you to eat breakfast every day? ________________________

SKIP LOGIC IF 24=No, and I don't think I will try: Can you explain why you don't think you will try to eat breakfast every day? ______________________________

25. Do you try to drink water instead of sugary drinks?
- ☐ Yes, I try and I do most of the time
- ☐ Yes, I try, but it is difficult
- ☐ No, but I will try in the future
- ☐ No, and I don't think I will try

SKIP LOGIC IF 25=Yes, I try and I do most of the time: What do you think helps you be successful at drinking water instead of sugary drinks? _______________

SKIP LOGIC IF 25=Yes, but it's difficult: Can you explain what makes it difficult to drink water instead of sugary drinks? __________________________________

SKIP LOGIC IF 25=No, but I will try it in the future: What do you think would make it easier for you to drink water instead of sugary drinks? _____________

SKIP LOGIC IF 25=No, and I don't think I will try: Can you explain why you don't think you will try to drink water instead of sugary drinks? ___________________

26. Do you try to eat mostly plant-based foods?
- ☐ Yes, I try and I do most of the time
- ☐ Yes, I try, but it is difficult
- ☐ No, but I will try in the future
- ☐ No, and I don't think I will try

SKIP LOGIC IF 26=Yes, I try and I do most of the time: What do you think helps you be successful at eating mostly plant-based foods? ______________________

SKIP LOGIC IF 26=Yes, but it's difficult: Can you explain what makes it difficult to eat mostly plant-based foods? ____
SKIP LOGIC IF 26=No, but I will try it in the future: What do you think would make it easier for you to eat mostly plant-based foods? _____________________

SKIP LOGIC IF 26=No, and I don't think I will try: Can you explain why you don't think you will try to eat mostly plant-based foods? _________________________

27. Do you try to paint your plate with fruits and vegetables?
☐ Yes, I try and I do most of the time
☐ Yes, I try, but it is difficult
☐ No, but I will try in the future
☐ No, and I don't think I will try

SKIP LOGIC IF 27=Yes, I try and I do most of the time: What do you think helps you be successful at painting your plate with fruits and vegetables? __________

SKIP LOGIC IF 27=Yes, but it's difficult: Can you explain what makes it difficult to paint your plate with fruits and vegetables? ______________________________

SKIP LOGIC IF 27=No, but I will try it in the future: What do you think would make it easier for you to paint your plate with fruits and vegetables? __________

SKIP LOGIC IF 27=No, and I don't think I will try: Can you explain why you don't think you will try to paint your plate with fruits and vegetables? ______________

28. Do you try to cook and eat at home?
☐ Yes, I try and I do most of the time
☐ Yes, I try, but it is difficult
☐ No, but I will try in the future
☐ No, and I don't think I will try

SKIP LOGIC IF 28=Yes, I try and I do most of the time: What do you think helps you be successful at cooking and eating at home? _________________________

SKIP LOGIC IF 28=Yes, but it's difficult: Can you explain what makes it difficult to cook and eat at home? ____

SKIP LOGIC IF 28=No, but I will try it in the future: What do you think would make it easier for you to cook and eat at home? __________________________

SKIP LOGIC IF 28=No, and I don’t think I will try: Can you explain why you don’t think you will try to cook and eat at home? ________________________________

29. Thinking about all of the CHEF classes you took: How did you like the CHEF classes overall?
- ☐ I liked them a lot
- ☐ I liked them
- ☐ I don't know if I liked them or not
- ☐ I did not like them
- ☐ I did not like them at all

SKIP LOGIC IF 29=I liked them a lot/I liked them: Please tell us what you liked about the classes: ____

SKIP LOGIC IF 29=I did not like them/I did not like them at all: Please tell us what you think can be made better about the classes: ____________________

30. Would you recommend the CHEF classes to your friends?
- ☐ Yes
- ☐ Maybe
- ☐ No
